# Supplementary material for: A simple liquid 3D cell culture paradigm models oxidative mitochondrial metabolism of epithelial breast cancer cells with relevance for lung metastases
Source: bioRxiv. 2025 Aug 27:2025.08.24.671623. Preprint. [Version 1] doi: 10.1101/2025.08.24.671623 (PMC12407794; doi:10.1101/2025.08.24.671623)
Supplement: Supplement 1 [file media-1.pdf]

SUPPLEMENTAL FIGURES AND LEGENDS for

A simple liquid 3D cell culture paradigm models oxidative mitochondrial metabolism of epithelial breast cancer cells with relevance for lung metastases

Kuppusamy Balamurugan[a*], Melissa R. Mikolaj[b,c], Jonathan M. Weiss[d], Ronald Holewinski[e], Xia Xu[e], Yu Fan[f], Lois McKennett[g], Christopher W. Dell1[b,c], Shikha Sharan[a], Duncan Donohue[h], Shashikala Ratnayake[f], Qingrong Chen[f], Daoud Meerzaman[f], Thorkel Andresson[e], Daniel W. McVicar[d], Kedar Narayan[b,c], and Esta Sterneck[a*]

[a] Laboratory of Cell and Developmental Signaling; Center for Cancer Research, National Cancer Institute, Frederick, MD 21702, USA.
[b] CCR Volume Electron Microscopy (CVEM), Center for Cancer Research, National Cancer Institute.
[c] Cancer Research Technology Program, Frederick National Laboratory for Cancer Research, Frederick, MD 21702, USA.
[d] Cancer Innovation Laboratory, Center for Cancer Research, National Cancer Institute, Frederick MD 21702, USA.
[e] Protein Characterization Laboratory, Cancer Research Technology Program, Leidos Biomedical Research Inc., Frederick National Laboratory for Cancer Research, Frederick, MD 21702, USA.
[f] Computational Genomics and Bioinformatics Branch, Center for Biomedical Informatics & Information Technology, National Cancer Institute, National Institutes of Health, Rockville, MD 20850, USA.
[g] Laboratory of Animal Sciences Program, Leidos Biomedical Research Inc., Frederick National Laboratory for Cancer Research, Frederick, MD 21702, USA.
[h] Statistical Consulting and Scientific Programming Group, Computer and Statistical Services, Data Management Services, Inc (a BRMI company), National Cancer Institute, Frederick, MD 21702, USA.

*Corresponding authors:
Esta Sterneck, National Cancer Institute, 1050 Boyles Street, Frederick, MD 21702-1201
sternecg@mail.nih.gov
Balamurugan Kuppusamy, National Cancer Institute, 1050 Boyles Street, Frederick, MD 21702-1201
kuppusamyb@nih.gov

SUPPLEMENTAL FIGURE LEGENDS

Figure S1. Single cell mRNA Sequencing of SUM149 and IBC-3 cells from 2D, SphC, and EmC
UMAP clusters of scRNA-Seq data derived from (A) SUM149 and (B) IBC-3 cells after 3 days in the indicated culture conditions, along with tables showing the number of cells per cluster and condition. Data represent the combination of two biological replicates each.

Figure S2. Ultrastructural analysis of SUM149 mitochondria, nuclei and lipid droplets

A. Scanning electron microscope images showing mitochondria under indicated conditions. Images taken in FIJI at 25% zoom with 5 µm x 2 µm box. Pixel size = 5 nm.
B. Scanning electron microscope images of nuclei as in A. Scale bar = 2 µm.
C. Morphometric data of nuclei in cells cultured as indicated (n = 50-68, mean).
D. Morphometric data of mitochondria in cells cultured as indicated (n = 1451-1995).
E. Scanning electron microscope images of lipid droplets LDs (as identified in the image on the left) in cells cultured as indicated. Pixel size=5 nm.
F. Morphometric data of lipid droplets in cells cultured as indicated (n = 131-654).
   ****P*<0.0001, *****P*<0.00001, n.s. not significant.

Figure S3. Proteomic and metabolic analysis reveals differential cell adaptations to 3D culture.

A. PCA and box plots of proteomic data (Supplemental File 5) from SUM149 cells in different culture conditions and xenograft tumor tissue (n = 3).
B. Volcano plots of the data in panel A.
C. Ingenuity Pathway Analysis of protein expression data as in panels A-B. Arrows point to pathways discussed in the Results.
D. Intracellular pyruvate, glutamate and NADH concentrations in SUM149 cells cultured for 3 days in SphC and EmC (n = 4).
E. Basal and maximal OCR in IBC-3 cells after 3 days under the indicated conditions, representative data from 3 time points and 3 technical replicates each.
F. Basal ECAR in IBC-3 cells, representative data from 3 time points and 3 technical replicates each.
G. Basal OCR and ECAR from MDA-MB-468 cells, representative data from 3 time points and 3 technical replicates each.
   Data are mean ± SEM,**P*<0.05, ***P*<0.01, ****P*<0.001, *****P*<0.0001, n.s., not significant.

Figure S4. Cells in EmC are sensitive to OXPHOS and ID1/ID3 inhibitors.

A. Percent PI positive cells in EmC treated with different doses of IACS-010579 for 72 h.
B. Percent PI positive cells in SphC treated with different doses of DCA for 72 h.
C. Bright filed images of SUM149 and MDA-MB-468 cells in EmC before and after treatment with ID1/ID3 inhibitor, AGX51. Scale bar=1 mm. Inset shows high magnification to visualize fraying periphery of the embolus in the presence of AGX51 of SUM159 cells.
   Data are mean ± SEM,**P*<0.05, ***P*<0.01, ****P*<0.001, n.s., not significant.

Figure S1

A. **SUM149**

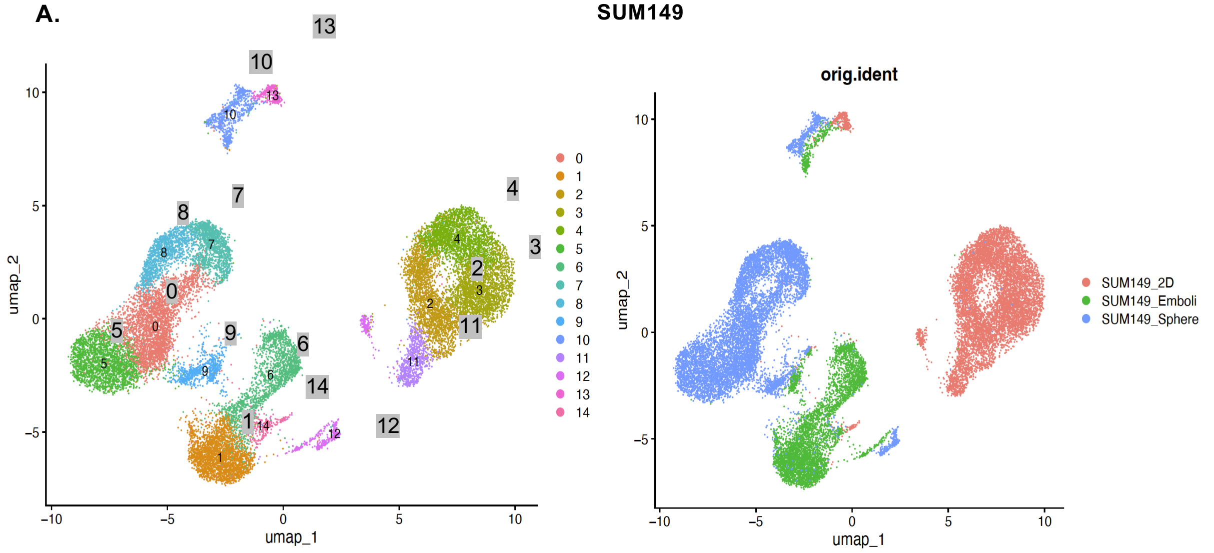

group	0	1	2	3	4	5	6	7	8	9	10	11	12	13	14
SUM149_2D	7	4	2408	2309	2253	2	18	4	2	33	15	725	153	277	64
SUM149_Emboli	6	2486	0	0	2	8	1818	3	3	259	294	1	103	17	164
SUM149_Sphere	3022	36	1	2	1	2225	33	1269	1269	468	444	3	199	7	13

B. **IBC-3**

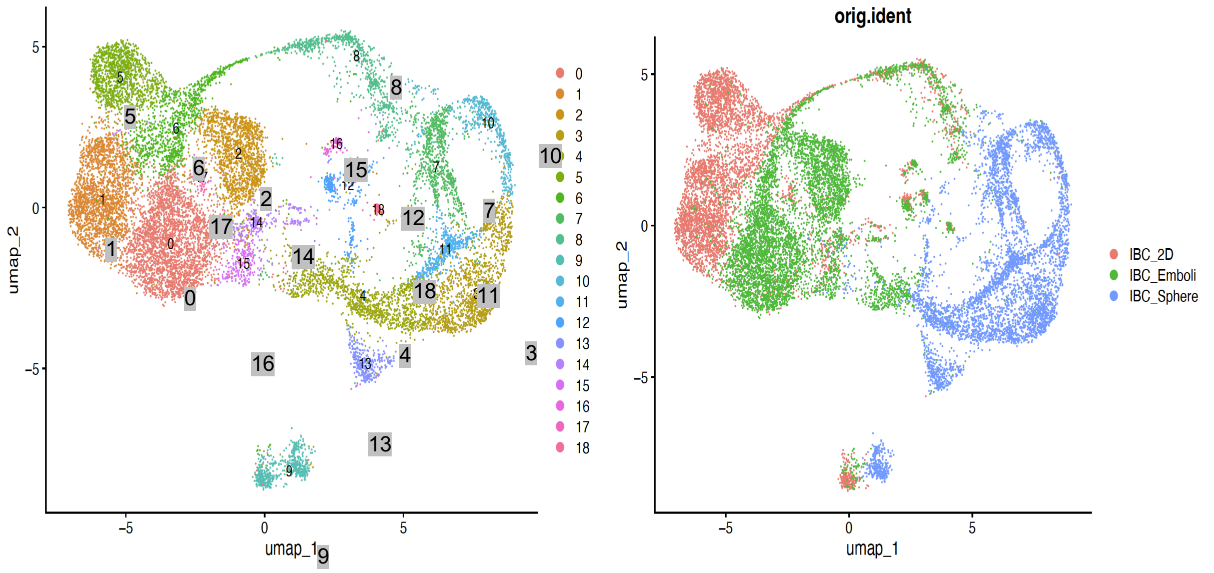

group	0	1	2	3	4	5	6	7	8	9	10	11	12	13	14	15	16	17	18
IBC_2D	115	2135	48	3	1	1251	563	9	294	209	7	0	113	0	104	64	51	50	33
IBC_Emboli	2745	36	1507	8	376	8	525	24	486	57	14	2	126	12	137	188	47	35	35
IBC_Sphere	5	0	1	1431	883	0	2	887	21	300	501	519	108	303	24	6	0	1	0

Figure S2

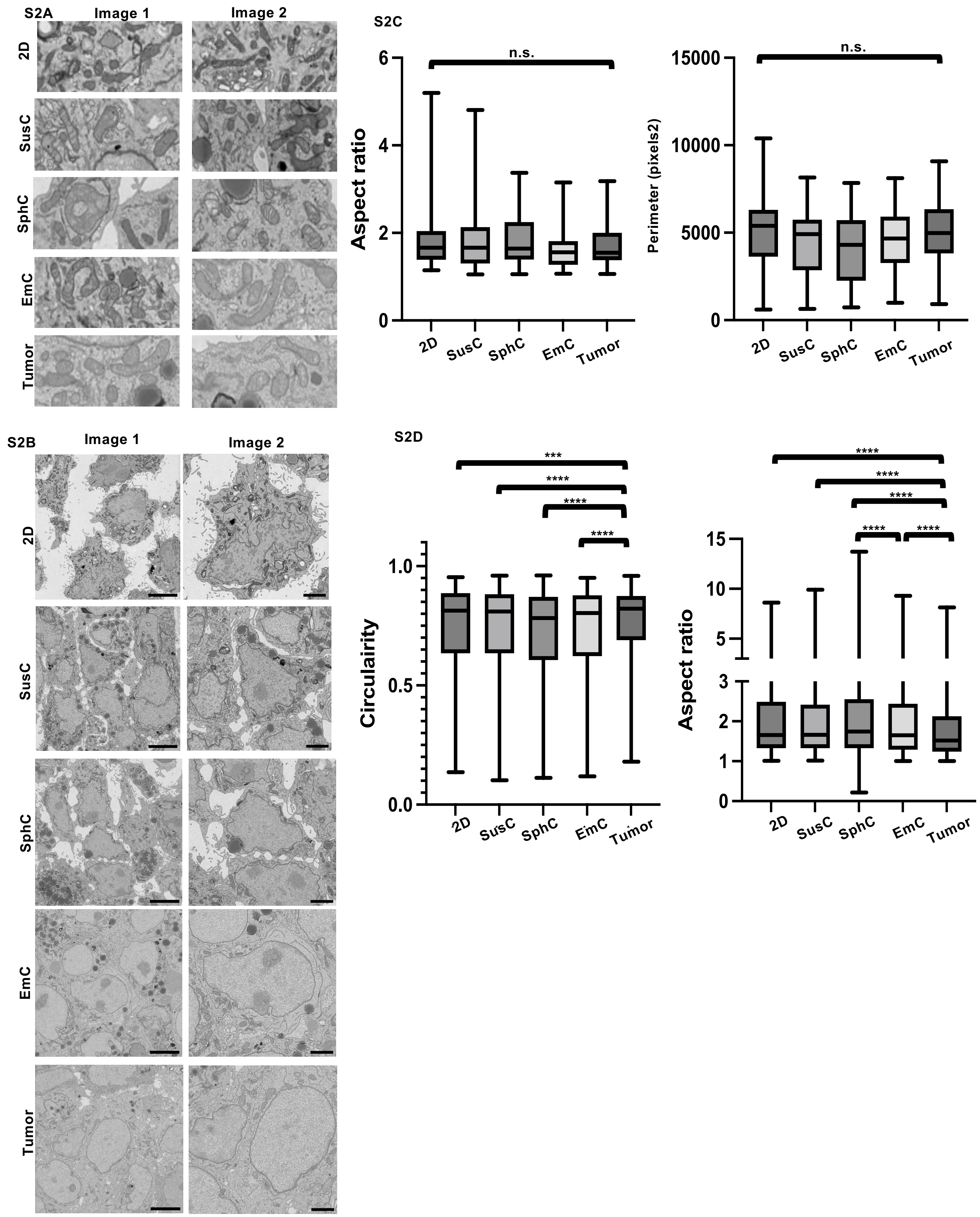
S2A
Image 1
Image 2
2D
SusC
SphC
EmC
Tumor
S2C
n.s.
Aspect ratio
Perimeter (pixels2)
0
2
4
6
5000
10000
15000
2D
SusC
SphC
EmC
Tumor
S2B
Image 1
Image 2
2D
SusC
SphC
EmC
Tumor
S2D
***
****
Circulairity
0.0
0.5
1.0
Aspect ratio
0
1
2
3
5
10
15
2D
SusC
SphC
EmC
Tumor

Figure S2

S2E

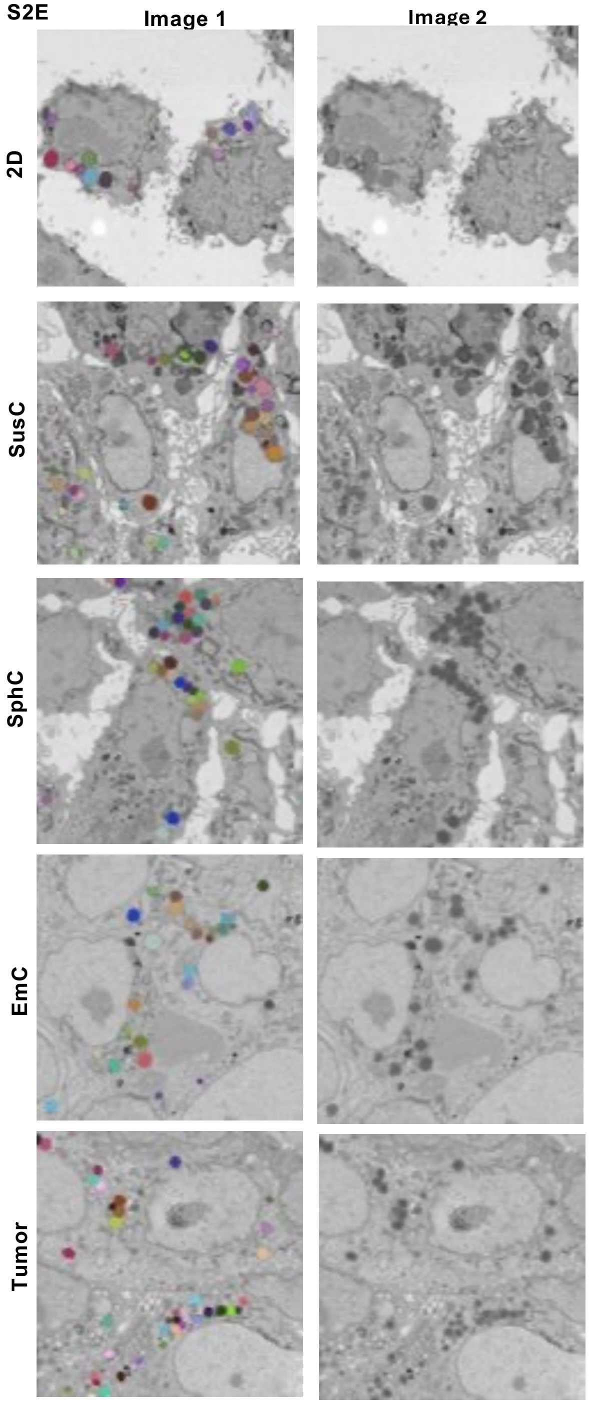

S2F

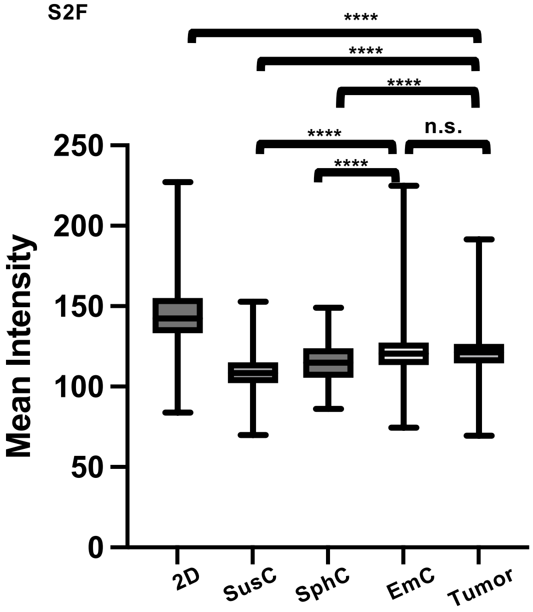

Figure S3

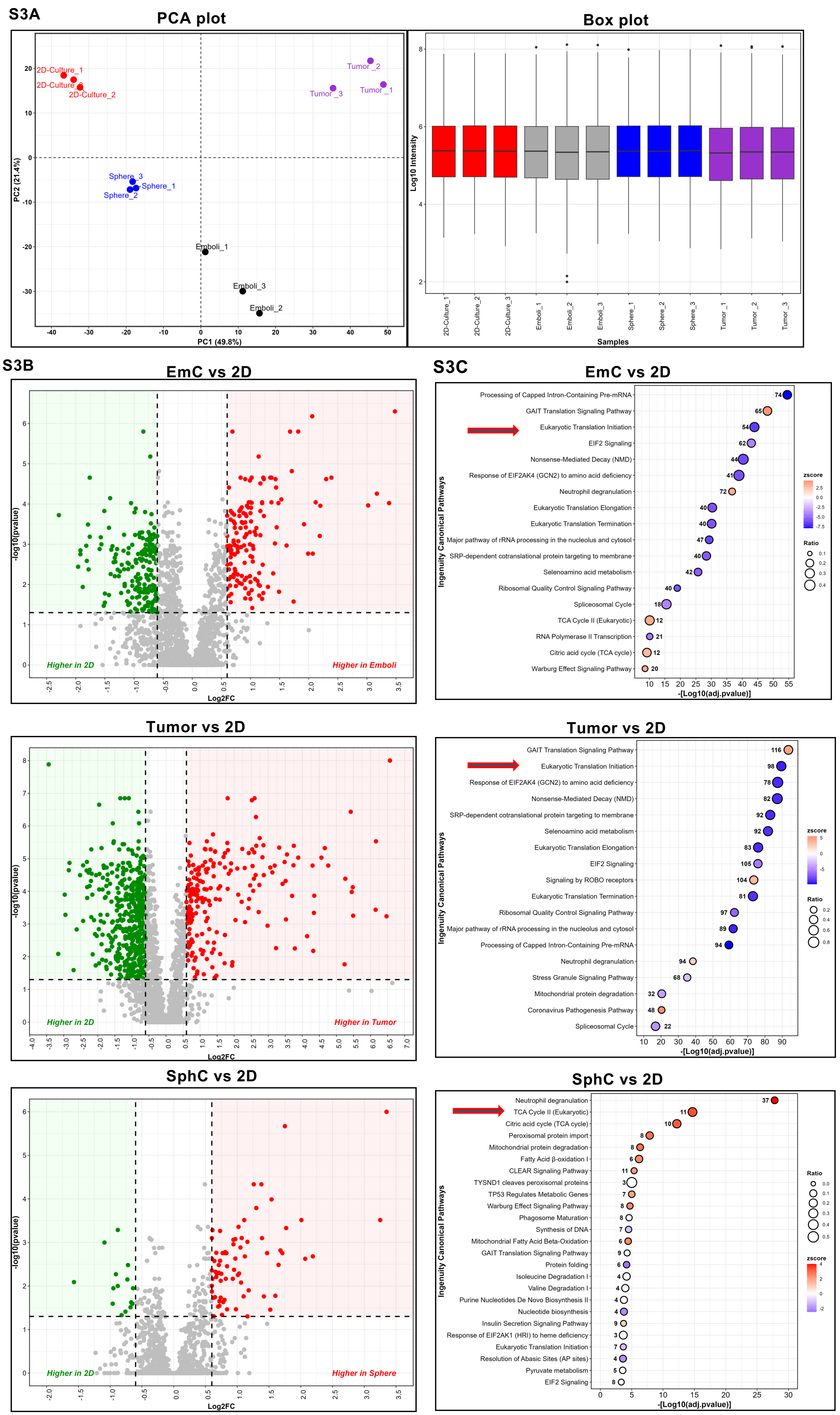

S3A
PCA plot
2D-Culture_1
2D-Culture_3
2D-Culture_2
Tumor_2
Tumor_3
Tumor_1
Sphere_3
Sphere_1
Sphere_2
Emboli_1
Emboli_3
Emboli_2
PC1 (49.8%)
PC2 (21.4%)
Box plot
Log10 Intensity
Samples
2D-Culture_1
2D-Culture_2
2D-Culture_3
Emboli_1
Emboli_2
Emboli_3
Sphere_1
Sphere_2
Sphere_3
Tumor_1
Tumor_2
Tumor_3
S3B
EmC vs 2D
-log10(pvalue)
Log2FC
Higher in 2D
Higher in Emboli
S3C
EmC vs 2D
Ingenuity Canonical Pathways
Processing of Capped Intron-Containing Pre-mRNA
GAIT Translation Signaling Pathway
Eukaryotic Translation Initiation
EIF2 Signaling
Nonsense-Mediated Decay (NMD)
Response of EIF2AK4 (GCN2) to amino acid deficiency
Neutrophil degranulation
Eukaryotic Translation Elongation
Eukaryotic Translation Termination
Major pathway of rRNA processing in the nucleolus and cytosol
SRP-dependent cotranslational protein targeting to membrane
Selenoamino acid metabolism
Ribosomal Quality Control Signaling Pathway
Spliceosomal Cycle
TCA Cycle II (Eukaryotic)
RNA Polymerase II Transcription
Citric acid cycle (TCA cycle)
Warburg Effect Signaling Pathway
-[Log10(adj.pvalue)]
zscore
Ratio
Tumor vs 2D
-log10(pvalue)
Log2FC
Higher in 2D
Higher in Tumor
Tumor vs 2D
Ingenuity Canonical Pathways
GAIT Translation Signaling Pathway
Eukaryotic Translation Initiation
Response of EIF2AK4 (GCN2) to amino acid deficiency
Nonsense-Mediated Decay (NMD)
SRP-dependent cotranslational protein targeting to membrane
Selenoamino acid metabolism
Eukaryotic Translation Elongation
EIF2 Signaling
Signaling by ROBO receptors
Eukaryotic Translation Termination
Ribosomal Quality Control Signaling Pathway
Major pathway of rRNA processing in the nucleolus and cytosol
Processing of Capped Intron-Containing Pre-mRNA
Neutrophil degranulation
Stress Granule Signaling Pathway
Mitochondrial protein degradation
Coronavirus Pathogenesis Pathway
Spliceosomal Cycle
-[Log10(adj.pvalue)]
zscore
Ratio
SphC vs 2D
-log10(pvalue)
Log2FC
Higher in 2D
Higher in Sphere
SphC vs 2D
Ingenuity Canonical Pathways
Neutrophil degranulation
TCA Cycle II (Eukaryotic)
Citric acid cycle (TCA cycle)
Peroxisomal protein import
Mitochondrial protein degradation
Fatty Acid β-oxidation I
CLEAR Signaling Pathway
TYSND1 cleaves peroxisomal proteins
TP53 Regulates Metabolic Genes
Warburg Effect Signaling Pathway
Phagosome Maturation
Synthesis of DNA
Mitochondrial Fatty Acid Beta-Oxidation
GAIT Translation Signaling Pathway
Protein folding
Isoleucine Degradation I
Valine Degradation I
Purine Nucleotides De Novo Biosynthesis II
Nucleotide biosynthesis
Insulin Secretion Signaling Pathway
Response of EIF2AK1 (HRI) to heme deficiency
Eukaryotic Translation Initiation
Resolution of Abasic Sites (AP sites)
Pyruvate metabolism
EIF2 Signaling
-[Log10(adj.pvalue)]
Ratio
zscore

Figure S3

S3D

SUM149

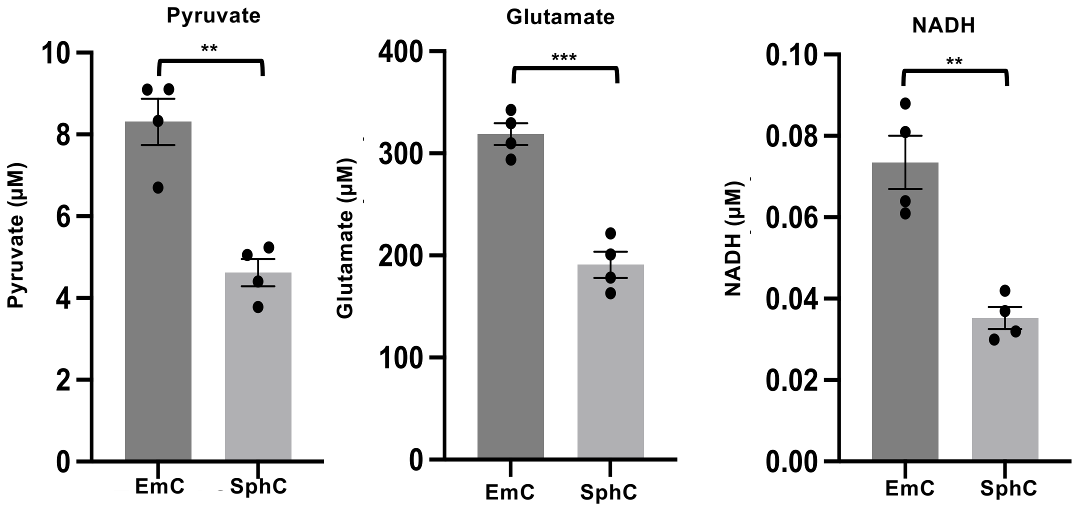

S3E

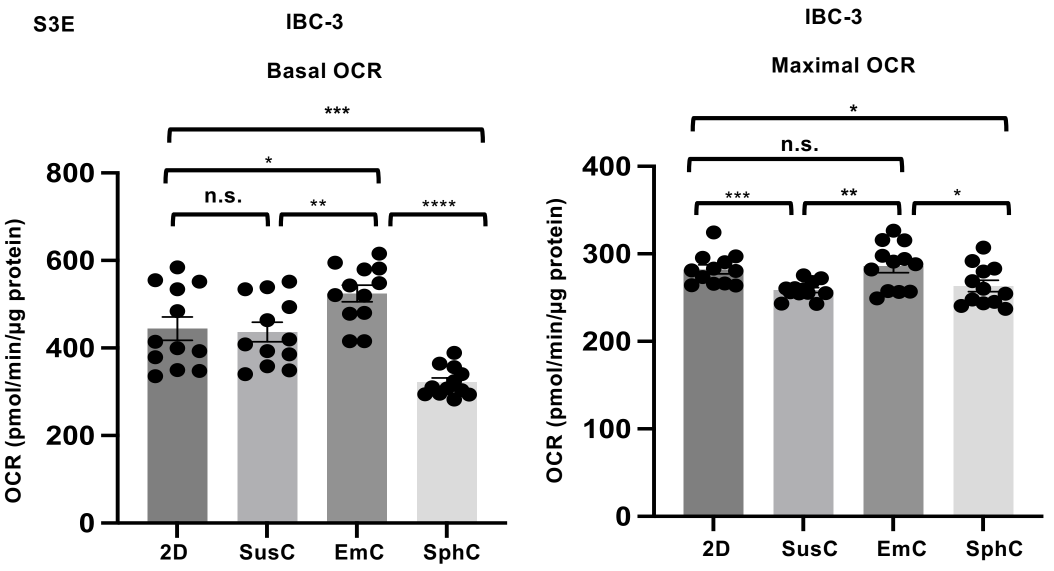

S3F

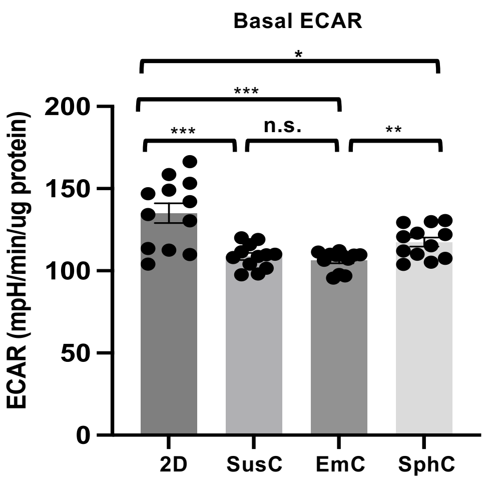

S3G

MDA-MB-468

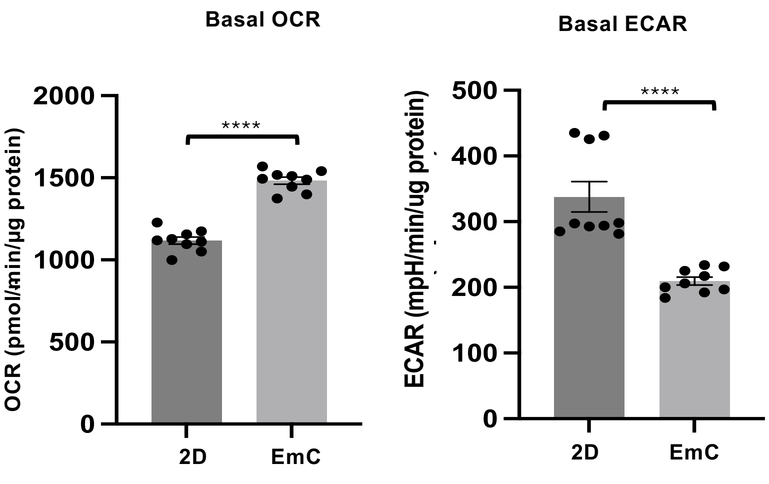

Figure S4

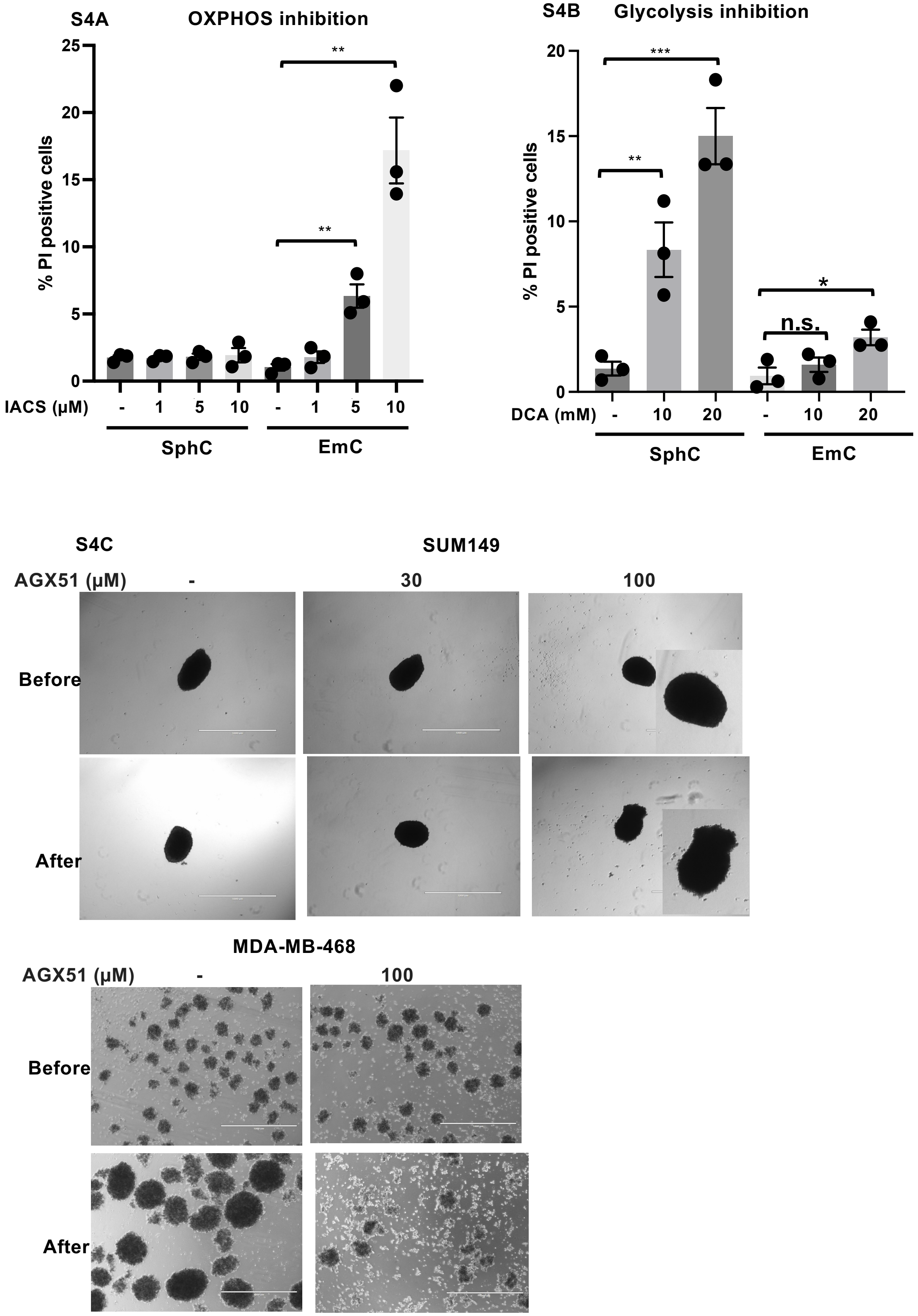
S4A
OXPHOS inhibition
% PI positive cells
0
5
10
15
20
25
**
**
IACS (μM)
-
1
5
10
-
1
5
10
SphC
EmC
S4B
Glycolysis inhibition
% PI positive cells
0
5
10
15
20
***
**
*
n.s.
DCA (mM)
-
10
20
-
10
20
SphC
EmC
S4C
SUM149
AGX51 (μM)
-
30
100
Before
After
MDA-MB-468
AGX51 (μM)
-
100
Before
After
